# Apoptosis is Induced in Cancer Cells via the Mitochondrial Pathway by the Novel Xylocydine-Derived Compound JRS-15

**DOI:** 10.3390/ijms14010850

**Published:** 2013-01-04

**Authors:** Chao Sun, Xiao-Xi Guo, Dan Zhu, Chuan Xiao, Xiao Bai, Yang Li, Zhuo Zhan, Xiang-Long Li, Zhi-Guang Song, Ying-Hua Jin

**Affiliations:** 1 Key Laboratory for Molecular Enzymology and Engineering of the Ministry of Education, College of Life Science, Jilin University, Changchun 130012, China; E-Mails: chaosun10@hotmail.com (C.S.); gxxzmcn@me.com (X.-X.G.); zhudanwjs@foxmail.com (D.Z.); liyang915@jlu.edu.cn (Y.L.); fred365@live.cn (Z.Z.); xianglong.lee@gmail.com (X.-L.L.); 2 College of Chemistry, Jilin University, Changchun 130021, China; E-Mail: xiaochuan0202@126.com; 3 Norman Bethune College of Medicine, Jilin University, Changchun 130021, China; E-Mail: baixiao0423@126.com

**Keywords:** apoptosis, Bax, Bak, Bcl-xL, cell cycle, cytochrome *c*, JRS-15, Smac, XIAP

## Abstract

The novel compound JRS-15 was obtained through the chemical modification of xylocydine. JRS-15 exhibited much stronger cytotoxic and pro-apoptotic activity than its parent compound in various cancer cell lines, with IC_50_ values in HeLa, HepG2, SK-HEP-1, PC-3M and A549 cells ranging from 12.42 to 28.25 μM. In addition, it is more potent for killing cancer than non-cancerous cells. Mechanistic studies showed that JRS-15 treatment arrested cell cycle at the G1/S phase, which further triggered the translocation of Bax and Bak to the mitochondria, resulting in mitochondrial membrane potential (MMP) depolarization and the subsequent release of cytochrome *c* and the second mitochondria-derived activator of caspase (Smac). The sequential activation of caspase-9 and caspase-3/7 and the cleavage of poly (ADP-ribose) polymerase (PARP) were observed following these mitochondrial events. Caspase-8, an initiator caspase that is required to activate the membrane receptor-mediated extrinsic apoptosis pathway was not activated in JRS-15-treated cells. Further analysis showed that the levels of the anti-apoptotic proteins Bcl-xL and XIAP were significantly reduced upon JRS-15 treatment. Furthermore, the caspase-9 inhibitor z-LEHD-fmk, the pan-caspase inhibitor z-VAD-fmk, and Bcl-xL or XIAP overexpression all effectively prevented JRS-15-induced apoptosis. Taken together, these results indicate that JRS-15 induces cancer cell apoptosis by regulating multiple apoptosis-related proteins, and this compound may therefore be a good candidate reagent for anticancer therapy.

## 1. Introduction

Cancer is among the most fatal diseases threatening humans worldwide [1]. The aberrant regulation of apoptosis is essential for cancer initiation and development [2]. Current cancer therapies mainly include gene therapy, immunotherapy, chemotherapy and radiation therapy, and these treatments primarily exert their anticancer effects by triggering cancer cell apoptosis [3].

Two major apoptotic pathways have been described: (1) the extrinsic pathway and (2) the intrinsic pathway [4]. In the extrinsic pathway, the activation of death receptors in the tumor necrosis factor (TNF) receptor superfamily, such as CD95 (Fas/Apo1), TNF-related apoptosis-inducing ligand receptor (TRAILR) and tumor necrosis factor receptor (TNFR) [5], results in receptor oligomerization and the recruitment of adaptor molecules, namely, Fas-associated death domain (FADD) and caspase-8. Upon recruitment, caspase-8 becomes activated and initiates apoptosis by directly cleaving the downstream effector caspase-3/7 [6–8]. The intrinsic pathway is initiated by the release of pro-apoptotic factors such as cytochrome *c*, Smac and apoptosis-inducing factor (AIF) from mitochondria into the cytosol [9–12]. In the cytosol, cytochrome *c* promotes the formation of the apoptosome complex, activating the initiator caspase-9 and subsequent caspase cascades. The activities of these caspases are further amplified by Smac, which neutralizes the inhibitory effects of inhibitor of apoptosis proteins (IAPs), eventually resulting in cell apoptosis [13–15].

Bcl-2 family members play a crucial role in regulating mitochondrial outer membrane permeability (MOMP) and the initiation of the intrinsic apoptosis pathway. Bcl-2 family members include anti-apoptotic proteins (e.g., Bcl-2, Bcl-xL, Bcl-w and Mcl-1) and pro-apoptotic proteins, which are further subdivided into BH3-only proteins (e.g., Bim, Bid, and Bad) and multi-domain Bax-like proteins (e.g., Bax and Bak) that contain various BH domains [16,17]. In response to apoptotic stimuli, BH3-only proteins are activated, neutralize anti-apoptotic Bcl-2 family members, and directly or indirectly promote the translocation and oligomerization of Bax and/or Bak, which results in changes in MOMP and the release of cytochrome *c* and Smac from the mitochondria [18–21]. The anti-apoptotic Bcl-2 family members can directly bind and inhibit the pro-apoptotic Bcl-2 family members to prevent apoptosis [22–24].

Xylocydine is a novel cyclin-dependent kinase (cdk) inhibitor that induces apoptosis in hepatocellular carcinoma (HCC) cells by inhibiting Cdk1, Cdk2, Cdk7, and Cdk9 activities [25,26]. However, its pro-apoptotic activity is weak, and it is cytotoxic only in SNU-354 and SNU-709 cells.

JRS-15 is a novel derivative of xylocydine. The objective of this study was to evaluate its pro-apoptotic effects on multiple human cancer cells, and investigate the underlying mechanisms. Here, we show that JRS-15, functions as a broad-range anticancer agent by effectively triggering apoptosis than xylocydine. Mechanistic studies in HeLa cells demonstrate that JRS-15 triggers the intrinsic apoptosis pathway.

## 2. Results

### 2.1. Synthesizing a Novel Compound JRS-15

**Figure 1:**
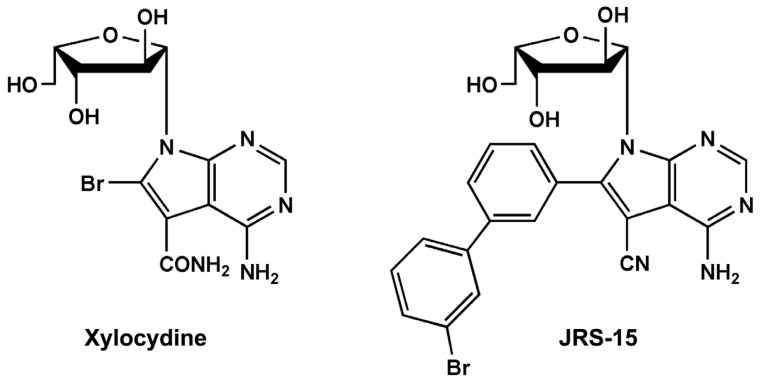
The molecular structures of xylocydine and JRS-15. Xylocydine is 4-amino-6-bromo-7-(β-l-xylofuranosyl)pyrrolo[2,3-*d*]pyrimidine-5-carboxamide. JRS-15 is 4-amino-6-(3-(3-bromophenyl)phenyl)-7-(β-l-xylofuranosyl)pyrolo[2,3-*d*]pyrimidine-5-cyano.

In order to develop effective anticancer drugs, we designed and synthesized a series of 6-substituted xylocydine derivatives, and eventually obtained an active compound JRS-15 (Figure 1). This work based on the crystal structures of Cdk2-cyclin A3-xylocydine ternary complex we had resolved (not published) and the conventional drug design principles [27–29]. The synthesis of 6-substituted xylocydine analogues compounds relied on palladium catalyzed Suzuki reactions of protected 4-amino-bromo-5-cyano-7-(2,3,5-tri-*O*-benzoyl-β-l-xylofuranosyl-)pyrrolo[2,3-*d*]pyrimidine **11** [30]. Details of the synthetic routes of JRS-15 were clearly described in the experimental section (Scheme I).

JRS-15 appeared as a solid powder, white in color; this is totally different from the original yellow color of xylocydine. In addition, both of these two compounds are highly soluble in DMSO.

### 2.2. JRS-15 Inhibits the Growth of Various Cancer Cells

**Figure 2:**
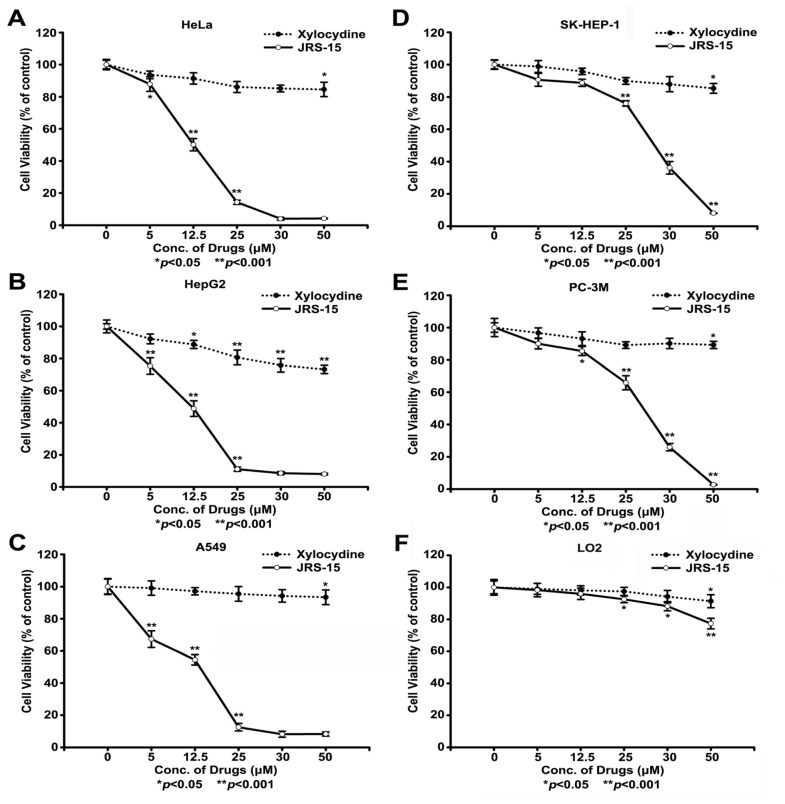
JRS-15 inhibits the growth of various human cancer cell lines more effectively than xylocydine. HeLa, HepG2, A549, SK-HEP-1, PC-3M, and LO2 cells were treated with 0.1% (*v*/*v*) DMSO (Control) or with the indicated concentrations of JRS-15 and xylocydine for 48 h. Cell viability was determined by MTT assay. All experiments were performed in triplicate (*p < 0.05, **p < 0.001).

To examine whether JRS-15 has a cytotoxic effect on human cancer cells, we assessed the viability of five different human cancer cell lines after treatment with JRS-15 using the MTT assay. The control groups were treated with 0.1% (*v*/*v*) DMSO, and no differences were found between 0.1% (*v*/*v*) DMSO and the negative controls. As shown in Figure 2 and Table 1, JRS-15 exhibited a broad-spectrum of growth inhibition and cytotoxicity against these cancer cell lines. JRS-15 strongly inhibited the growth of HeLa, HepG2 and A549 cells, with IC_50_ values ranging from 12.42 to 14.25 μM. It had moderate cytotoxic activity in SK-HEP-1 and PC-3M cells, with IC_50_ values of approximately 28.00 μM. Furthermore, the cytotoxic activity of JRS-15 was much greater than that of its parent compound xylocydine. Importantly, the variation in IC_50_ value of JRS-15 against different cell lines indicates that JRS-15 is more potent to kill cancer than non-cancerous cells.

### 2.3. JRS-15 Induces Apoptotic Cell Death in Various Cancer Cells

**Figure 3:**
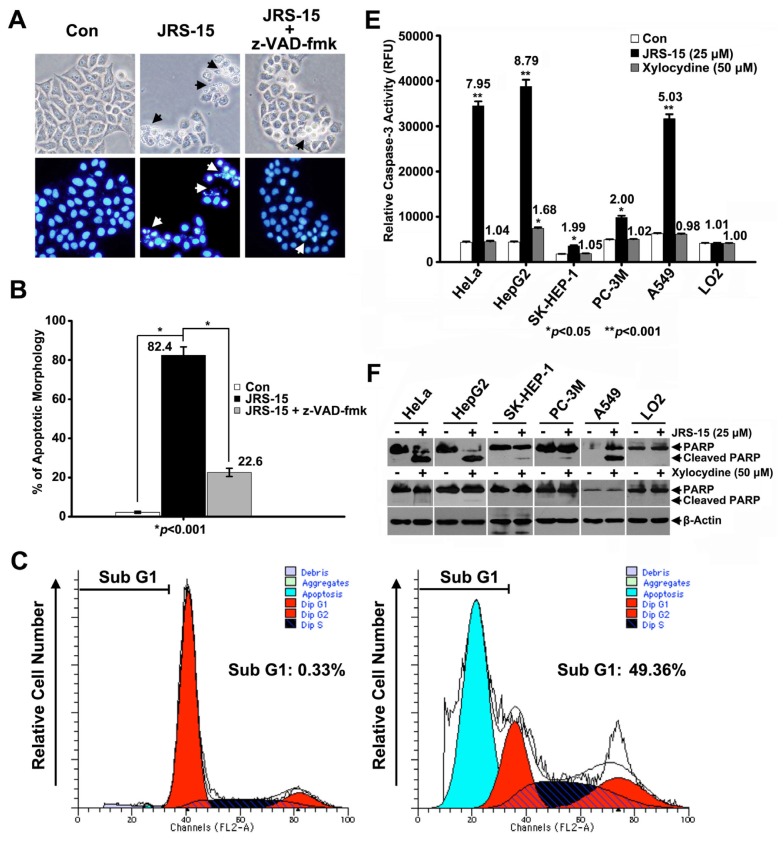

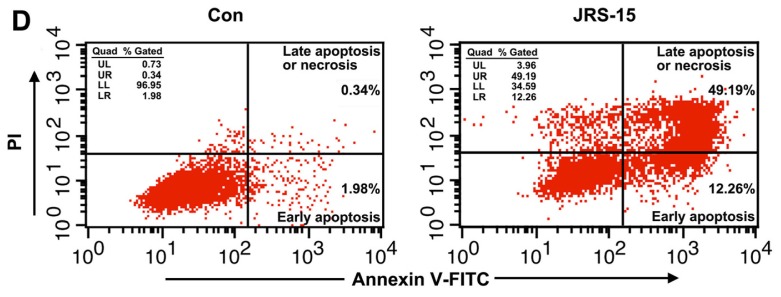
JRS-15 induces caspase-dependent apoptosis in cancer cells. (**A**) HeLa cells were pre-treated or not with 100 μM z-VAD-fmk, a pan-caspase inhibitor, for 2 h before they were treated with 25 μM JRS-15 for 24 h. The cells were stained with DAPI and imaged using a fluorescence microscope. The black arrows and white arrows indicate apoptotic bodies and chromatin condensation, respectively. (**B**) Quantitation of results shown in (A) (*p < 0.001). HeLa cells were treated or not with 25 μM JRS-15 for 24 h. Cells were stained with PI or Annexin V-FITC/PI, and the degree of apoptosis was determined by measuring (**C**) the area of the sub-G1 peak and (**D**) the population of Annexin V/PI-positive cells by flow cytometry. (**E**) Six different cell lines were treated or not with 25 μM JRS-15 or 50 μM xylocydine for 24 h, and their caspase-3 activities were measured (*p < 0.05, **p < 0.001). (**F**) Whole-cell lysates were analyzed for PARP and β-actin by immunoblotting. (The control groups were treated with 0.1% (*v*/*v*) DMSO, including the following assays.)

To examine whether JRS-15 decreased cell viability by inducing apoptosis, we analyzed the cells for morphological characteristics typical of apoptosis. Following 24 h of treatment with 25 μM JRS-15, over 82.4% of HeLa cells demonstrated characteristic apoptotic morphology, such as cell rounding and membrane blebbing (Figure 3A, top middle). In addition, DAPI staining showed that JRS-15 induced both chromatin condensation and nuclear fragmentation (Figure 3A, bottom middle). These morphological changes were dramatically blocked by pre-treatment of the cells with a pan-caspase inhibitor, z-VAD-fmk (Figure 3A, right panel). We then analyzed the cell cycle progression of HeLa cells after 24 h of treatment with 25 μM JRS-15. The fraction of cells with sub-G1 DNA content increased to 49.36% after JRS-15 treatment. In contrast, only 0.33% of cells in the control group had sub-G1 DNA content (Figure 3C). Similar effects were detected by measuring the fraction of Annexin V-FITC/PI-positive cells using flow cytometry. The result showed that over 12.26% early apoptosis and 49.19% late apoptosis appeared after cells treated with 25 μM JRS-15 for 24 h (Figure 3D). We then measured the cell-free caspase-3 activity in five cancer cell lines (HeLa, HepG2, SK-HEP-1, PC-3M and A549 cells) treated with 25 μM JRS-15 for 24 h. The results showed that the caspase-3 activities of the treated cells increased 1.99- to 8.79-fold in comparison to the control groups (Figure 3E). The effect of JRS-15 on caspase-3 activity in these cells was further evaluated by immunoblotting analysis to measure PARP (a specific substrate of caspase-3) cleavage, and similar effects on PARP cleavage were observed under these conditions (Figure 3F). However, the caspase-3 activities were not detected upon xylocydine treatment for 24 h, even at a concentration of 50 μM, except for HepG2 cells with slight elevation (Figure 3E,F). Similar effects were observed in HeLa cells upon xylocydine (75 μM, time gradient) treatment by kinetic study and immunoblot analysis on caspase-8, -9, and PARP (Figure S1). So in the following analysis on the dynamics of apoptosis induced by JRS-15, we didn’t perform the assays with xylocydine as a comparison. Importantly, the caspase-3 activity was not detected under 25 μM JRS-15 or 50 μM xylocydine treatment in LO2 cells (Figure 3E,F). In addition, the cleavages of caspase-8, -9 and PARP in LO2 cells were not observed, even at a concentration of 30 μM JRS-15 treatment for 24 h (Figure S2). In conclusion, JRS-15 induced cell death in multiple cancer cell lines by triggering apoptosis in a caspase-dependent manner. JRS-15 is more powerful than xylocydine, and exhibited selective at inducing apoptosis between cancer and normal liver cells.

### 2.4. JRS-15-Induced Apoptosis in HeLa Cells is Caspase-9-Dependent

**Figure 4:**
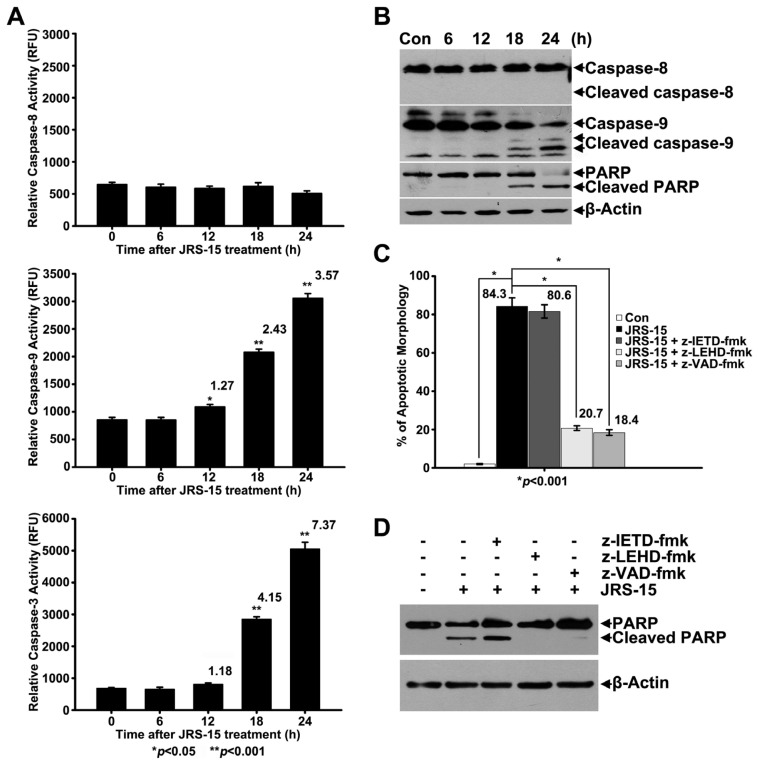
JRS-15-induced HeLa cell apoptosis depends on caspase-9 and caspase-3 activities. HeLa cells were treated or not with 25 μM JRS-15 for the indicated times. (**A**) Cell-free caspase-3, -8 and -9 activities were measured (*p < 0.01, **p < 0.001). (**B**) Whole-cell lysates were analyzed by immunoblotting for caspase-8, caspase-9, PARP and β-actin. HeLa cells were pre-treated or not with 100 μM z-IETD-fmk, a caspase-8 specific inhibitor, z-LEHD-fmk, a caspase-9 specific inhibitor, and z-VAD-fmk, a pan-caspase inhibitor for 2 h before treatment with 25 μM JRS-15 for 24 h. (**C**) Apoptotic cell death was measured by counting apoptotic cells, and (**D**) whole-cell lysates were analyzed by immunoblotting for PARP and β-actin.

To identify the caspase activation pathway in JRS-15-induced HeLa cell apoptosis, we investigated the activation kinetics of the initiator caspases-8 and -9 and the downstream effector caspase-3. The results showed that caspase-9 activity was slightly elevated in cells that had been treated with JRS-15 for 12 h. The activities of caspase-9 and caspase-3 were markedly upregulated after cells were treated with JRS-15 for 18 h and 24 h, whereas the caspase-8 activity remained unchanged at 24 h of treatment (Figure 4A). Similarly, immunoblot analysis demonstrated that caspase-9 and PARP cleavage was increased in a time-dependent manner when cells were treated with JRS-15 for 18 h or longer. However, no proteolytic activation of caspase-8 was detected (Figure 4B). Moreover, in HeLa cells, JRS-15-induced morphological changes were significantly blocked by pre-treatment with z-LEHD-fmk, a caspase-9 inhibitor, or z-VAD-fmk, a pan-caspase inhibitor (Figure 4C). Similarly, PARP cleavage was also inhibited in the cells pre-treated with caspase-9 or pan-caspase inhibitor but not in cells treated with z-IETD-fmk, a caspase-8 inhibitor (Figure 4D). These observations demonstrate that JRS-15-induced HeLa cell apoptosis depends on caspase-9 and caspase-3 activation.

### 2.5. JRS-15 Triggers the Mitochondrial Apoptotic Pathway in HeLa Cells

**Figure 5:**
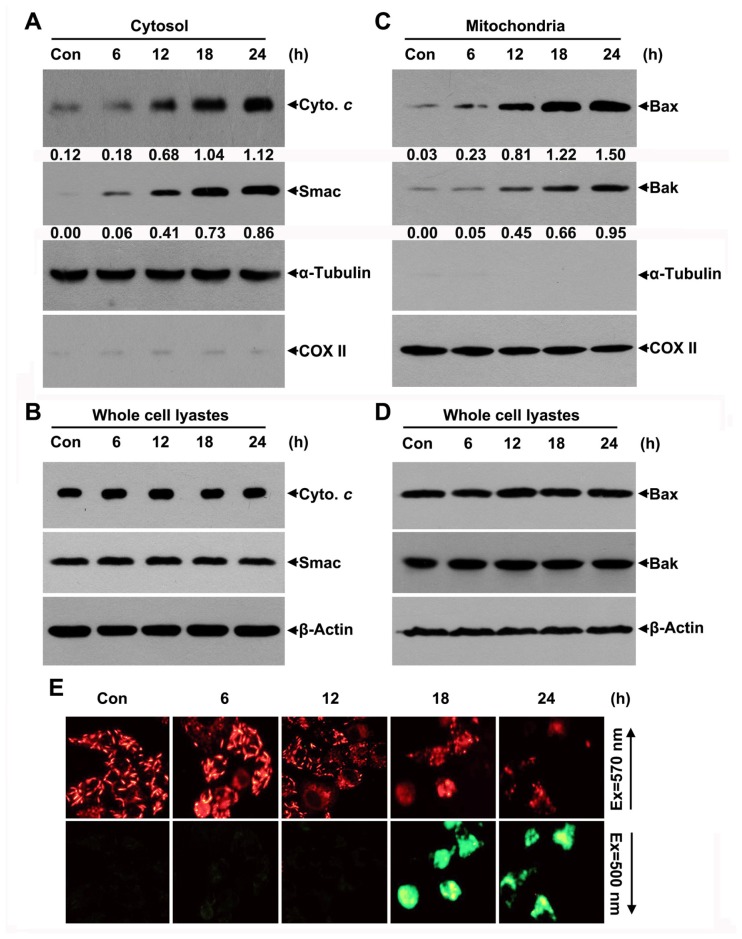
JRS-15 triggers the release of cytochrome *c* and Smac as well as Bax and Bak translocation during apoptosis. HeLa cells were treated or not with 25 μM JRS-15 for the indicated times. (**A**) Cytosolic fraction, (**C**) mitochondrial fraction, and (**B**,**D**) whole-cell lysates were analyzed by immunoblotting for cytochrome *c*, Smac, Bax, Bak, α-tubulin, β-actin, and COX II. The numbers below the bands of Cyto. *c*, Smac, Bax and Bak represent each band’s relative abundance to the loading controls, which were quantified by Image-Pro Plus software. (**E**) Cells were stained with a mitochondria-specific cation dye (MitoCapture). Images were captured using a fluorescence microscope with excitation wavelengths of 570 nm and 500 nm, respectively. Cells with normal, polarized mitochondria emit punctate red fluorescence, while depolarized mitochondrial membranes emit a diffuse green fluorescence.

Cytochrome *c* and Smac release from the mitochondrial inner membrane space into the cytosol has been shown to be a key event in the activation of caspase-9, which subsequently initiates a caspase cascade involving caspase-3 [9,13]. Because JRS-15-induced HeLa cell apoptosis is mediated by the caspase-9 and caspase-3 pathway (Figure 4), we investigated cytochrome *c* and Smac release in JRS-15-treated HeLa cells. Immunoblot analysis demonstrated that cytochrome *c* and Smac in the cytoplasmic fraction appeared at 12 h after JRS-15 treatment and gradually increased over time (Figure 5A). The protein levels of cytochrome *c* and Smac in the whole-cell fraction did not undergo any detectable changes (Figure 5B).

The Bcl-2 family proteins control MOMP, and therefore cytochrome *c* and Smac release, while the multi-domain Bcl-2 family members Bax and Bak serve as a necessary gateway for the release of apoptotic factors [17,18]. We assessed the translocation of Bax and Bak by immunoblot analysis. Bax and Bak proteins were detected in HeLa cell mitochondrial fractions after 12 h of treatment with JRS-15, and the levels of these proteins continued to increase in a time-dependent manner (Figure 5C). This increase was consistent with the data in Figure 5A. In contrast, the levels of Bax and Bak in whole-cell fractions did not change upon JRS-15 treatment (Figure 5D).

MMP depolarization assays were also performed. HeLa cells were treated or not with JRS-15 for the indicated times and stained with a mitochondria-specific cation dye (MitoCapture). The results showed that the intensity of bright red fluorescence, indicative of normal mitochondria, decreased at 12 h and diminished significantly at 18 h and 24 h after JRS-15 treatment (Figure 5E, top). In contrast, the intensity of diffuse green fluorescence, indicative of depolarized mitochondria, was inversely related to that of the red fluorescence in JRS-15-treated cells (Figure 5E, bottom). The close association of Bax and Bak translocation, MMP depolarization, and cytochrome *c* and Smac release with subsequent activation of caspase-9 clearly suggests that JRS-15-induced HeLa cell apoptosis occurs through the intrinsic mitochondrial apoptosis pathway.

### 2.6. JRS-15 Reduces Bcl-xL and XIAP Protein Levels in HeLa Cells

**Figure 6:**
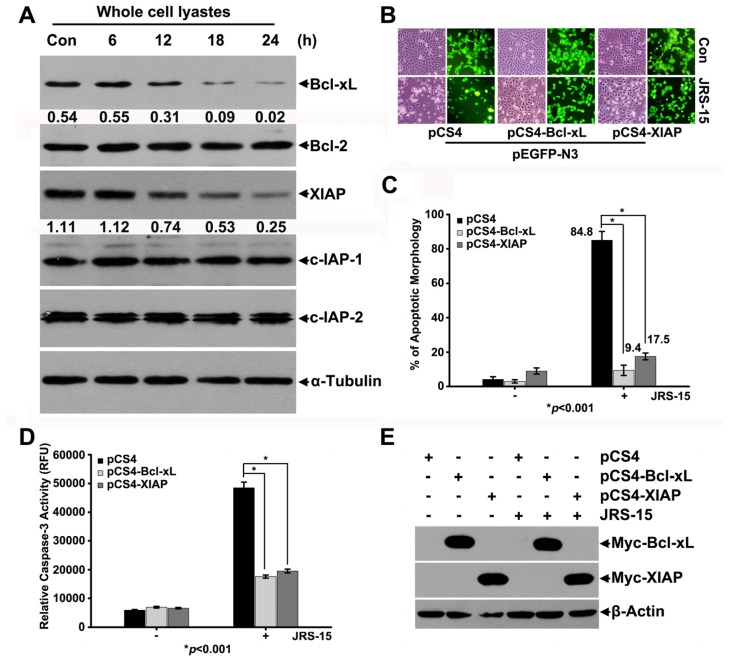
JRS-15 reduces Bcl-xL and XIAP protein levels, and Bcl-xL and XIAP overexpression prevents JRS-15-induced HeLa cell apoptosis. (**A**) HeLa cells were treated or not with 25 μM JRS-15 for the indicated times. Whole-cell lysates were analyzed by immunoblotting for Bcl-xL, Bcl-2, XIAP, c-IAP-1, c-IPA-2, and α-tubulin. The numbers below the bands of Bcl-xL and XIAP represent each band’s relative abundance to the loading controls, which were quantified by Image-Pro Plus software. HeLa cells were co-transfected with pEGFP-N3/pCS4, pEGFP-N3/pCS4-Bcl-xL, and pEGFP-N3/pCS4-XIAP. At 24 h post-transfection, cells were treated or not with 25 μM JRS-15 for an additional 24 h. (**B**) Images were captured by a fluorescence microscope. The bright field (left) and GFP fluorescent field (right) images represent the same areas. (**C**) The percentage of cells with apoptotic morphology was calculated by counting cells corresponding to (**B**) (*p < 0.001). (**D**) Caspase-3 activity in co-transfected cells was determined (*p < 0.001). (**E**) The protein levels of Bcl-xL and XIAP in co-transfected cells were analyzed by immunoblotting for c-Myc and β-actin.

We measured the levels of anti-apoptotic proteins to further understand the mechanisms underlying JRS-15-induced HeLa cell apoptosis. Immunoblot analysis in HeLa cells demonstrated that the levels of the anti-apoptotic molecules Bcl-xL and XIAP were downregulated at 12 h post-treatment, and their degradation was significantly increased at 18 h and 24 h after JRS-15 treatment. In contrast, the levels of other anti-apoptotic molecules, such as Bcl-2, c-IAP-1 and c-IAP-2, showed no detectable changes (Figure 6A). To further investigate whether JRS-15-induced HeLa cell apoptosis is due to Bcl-xL and XIAP downregulation, pEGFP-N3/pCS4, pEGFP-N3/pCS4-Bcl-xL, and pEGFP-N3/pCS4-XIAP were co-transfected into HeLa cells for 24 h followed by JRS-15 treatment of the transfected cells for an additional 24 h. At the end of the treatment period, cells with either normal or apoptotic morphology were identified by counting the GFP-positive cells. Our results indicated that following JRS-15 treatment the percentage of apoptotic cells amongst the Bcl-xL- and XIAP-overexpressing cells was significantly lower than in the control group (Figure 6B,C). Similarly, the caspase-3 activity was markedly attenuated in Bcl-xL- and XIAP-overexpressing cells under these conditions (Figure 6D). These data suggest that JRS-15-induced HeLa cell apoptosis is associated with the downregulation of anti-apoptotic proteins, Bcl-xL and XIAP. Moreover, overexpression of Bcl-xL and XIAP significantly inhibited JRS-15-induced HeLa cell apoptosis.

### 2.7. JRS-15 Induces Cell Cycle Arrest in HeLa Cells

**Figure 7:**
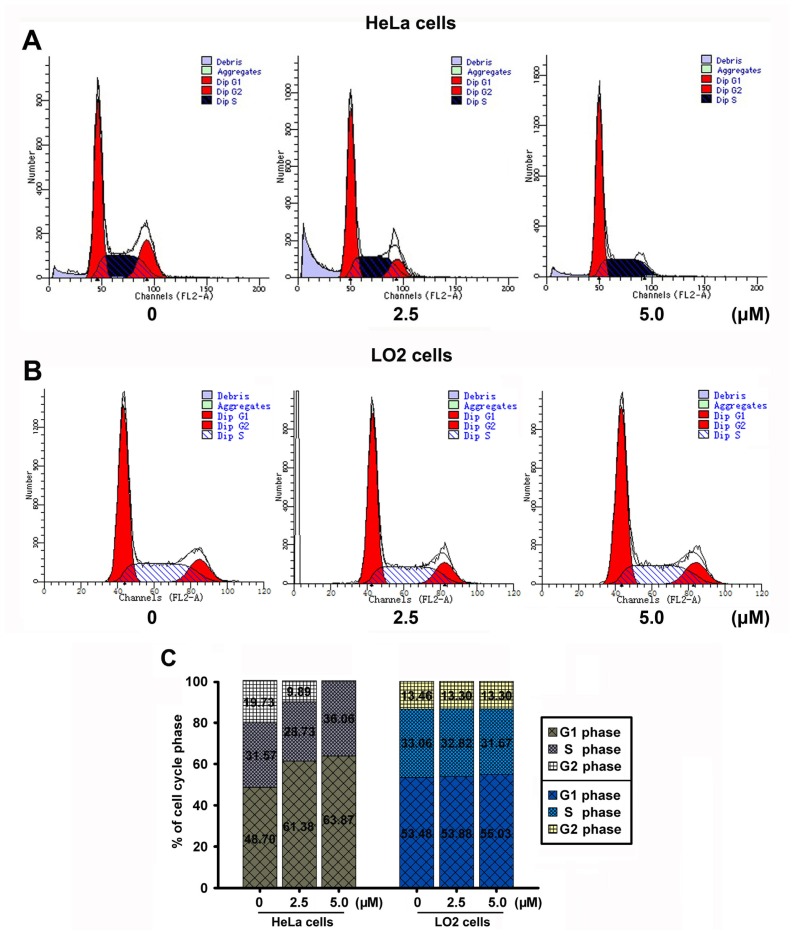
JRS-15 triggers cell cycle arrest in HeLa cells. (**A**,**B**) HeLa and LO2 cells were treated with 0.1% (*v*/*v*) DMSO (Control) or with the indicated concentrations of JRS-15 for 48 h. Cell cycle distributions were analyzed by a flow cytometry. (**C**) The percentage of G1, S and G2 phase shown in (**A**,**B**).

To gain an insight into the mechanism of the anti-proliferative activity of JRS-15, its effects on cell cycle distribution were determined in HeLa and LO2 cells. The results showed that JRS-15 strongly blocked the cell cycle at G1/S phase in HeLa cells under the low concentrations in a dose-dependent fashion (Figure 7A,C), whereas minimally influenced the normal liver LO2 cells under the same conditions (Figure 7B,C). As mentioned above, JRS-15 treatment with 25 μM could induce apoptosis in various cancer cell lines (Figure 3E,F). These observations suggest that JRS-15-induced HeLa cell apoptosis may start with cell cycle arrest, which triggers further apoptotic signaling.

### 2.8. Low Caspase-9 Expression in SK-HEP-1 Cells May Contribute to Their Resistance to JRS-15-Induced Apoptosis

**Figure 8:**
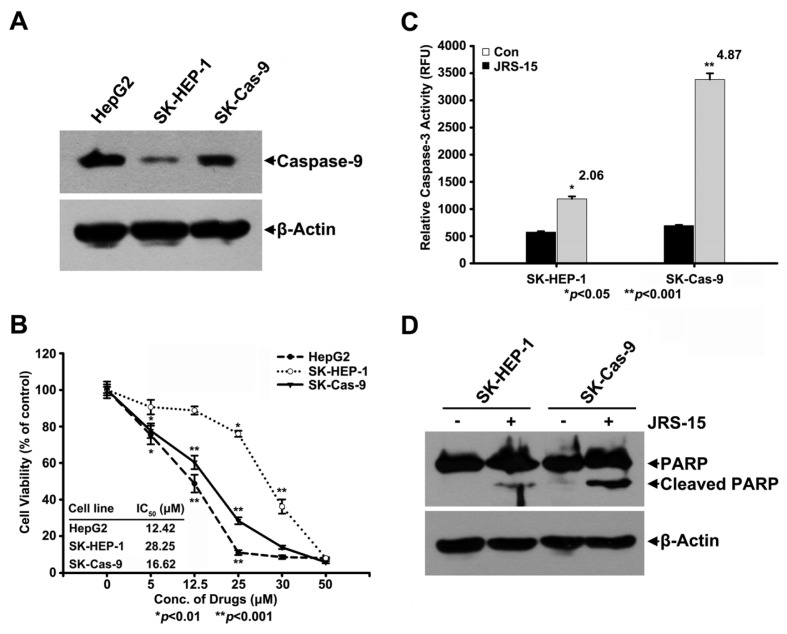
The overexpression of caspase-9 increases the sensitivity of JRS-15 in SK-HEP-1 cells. (**A**) The protein levels of caspase-9 were measured in HepG2, SK-HEP-1, and SK-Cas-9 cells by immunoblotting for caspase-9 and β-actin. (**B**) HepG2, SK-HEP-1, and SK-Cas-9 cells were treated with the indicated concentrations of JRS-15 for 48 h in triplicate experiments. Cell viability was determined by MTT assay (*p < 0.01, **p < 0.001). SK-HEP-1 and SK-Cas-9 cells were treated or not with 25 μM JRS-15 for 24 h, and (**C**) the caspase-3 activities of the treated cells were measured (*p < 0.05, **p < 0.001). (**D**) Whole-cell lysates were analyzed by immunoblotting for PARP and β-actin.

The data shown in Figure 2 and Table 1 suggest that the IC_50_ values of JRS-15 in SK-HEP-1 and PC-3M cells were higher than those in HeLa, HepG2, and A549 cells. In addition, the caspase-3 activity in SK-HEP-1 and PC-3M cells was only slightly elevated compared with that in the other three cell lines following 25 μM JRS-15 of treatment (Figure 3E). Similar results were obtained for PARP cleavage under the same conditions (Figure 3F). Interestingly, the IC_50_ value of JRS-15 was dramatically decreased, to 16.62 μM, in a SK-HEP-1 cell line that stable overexpressing caspase-9 (SK-Cas-9), and the cell viability trend was similar to that of HepG2 cells (Figure 8A,B). In addition, caspase-3 activity was significantly increased in SK-Cas-9 cells compared with SK-HEP-1 cells (Figure 8C). Similar effects were also observed in the PARP cleavage assay after treatment of cells with 25 μM JRS-15 (Figure 8D). These data suggest that the downregulation of caspase-9 in SK-HEP-1 may underlie the resistance of these cells to JRS-15-induced apoptosis.

## 3. Discussion

An increasing number of studies have implicated apoptosis as an important mechanism by which chemotherapeutic agents kill susceptible cells [3]. Previous reports have demonstrated that xylocydine is able to induce apoptosis in HCC cells, but its pro-apoptotic property is weak [26]. Here, we show that JRS-15, a novel compound which is derived from xylocydine by replacing the 6-Br position with 3-(3-bromophenyl)-phenyl (Figure 1), can effectively inhibits the cell growth on multiple cancer cell types by triggering apoptosis, such as cervical, hepatic, prostate and lung cancer cells, and its anti-proliferative effects are more powerful than xylocydine (Figures 2 and 3). Importantly, JRS-15 is less cytotoxic to the normal liver cells than cancerous cells (Figure 2), and is selective at inducing apoptosis between cancer and normal cells (Figure 3E,F and Figure S2). Moreover, cell cycle analysis indicates that JRS-15 minimally influences normal liver LO2 cells compared to HeLa cells under the same conditions (Figure 7).

**Figure 9:**
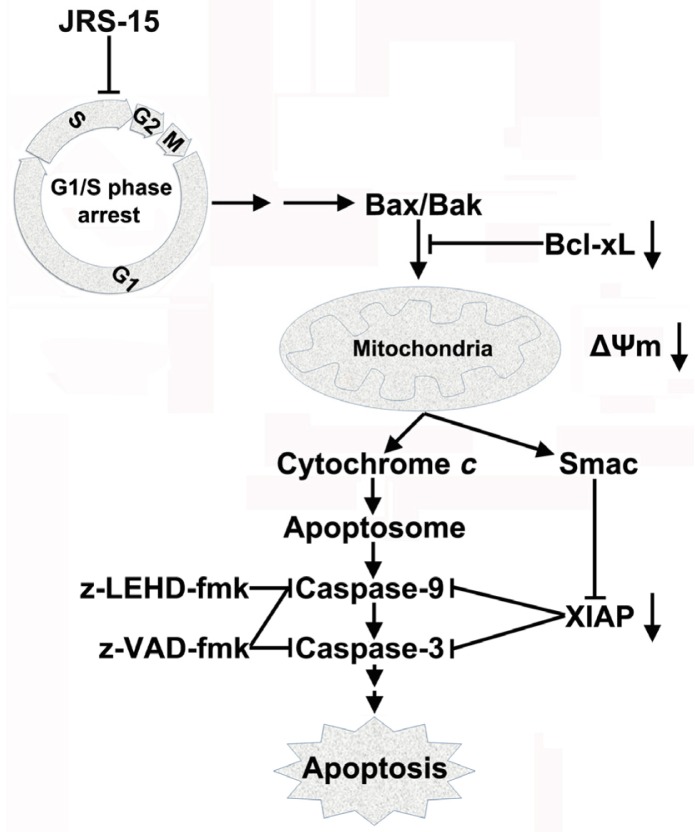
Schematic diagram showing the proposed apoptotic signaling pathways triggered by JRS-15 in HeLa cells. JRS-15 treatment blocks cell cycle at G1/S phase, which triggers the apoptotic signaling by step or multistep. In response to apoptotic stimuli, Bax and Bak, translocate to mitochondria, resulting in the depolarization of MMP and downstream release of cytochrome *c* and Smac, which is coincided with the downregulation of anti-apoptotic proteins, Bcl-xL and XIAP. These events all contribute to the subsequent activation of initiator caspase-9 and effector caspase-3, and eventually leading cell to an inevitable death.

To investigate the mechanisms of JRS-15-induced apoptosis, we selected HeLa cells as a model system. Here, we have proven that JRS-15 induces HeLa cell apoptosis by a mechanism that is functionally associated with cell cycle arrest, a series of mitochondrial events and caspase-3 activation. JRS-15 treatment at low concentrations resulted in strong G1/S phase cell cycle arrest (Figure 7). Under high concentrations of treatment, morphological determination, cell cycle distribution analysis, and Annexin V/PI analysis clearly demonstrated that JRS-15 induces caspase-dependent apoptosis (Figure 3). These observations show that JRS-15 triggered apoptosis may start with strongly blocking cell cycle. Further study showed that JRS-15-induced apoptosis involved the elevation of caspase-9 activity, which was detected at 12 h and that the activities of caspase-9 and caspase-3 were dramatically increased at 18 h after JRS-15 treatment (Figure 4A). This result was consistent with that of immunoblot analysis of caspase-9 and PARP cleavage under the same conditions (Figure 4B). As expected, the activation kinetics of initiator caspase-9 and the effector caspase-3 correlate well over time with that of the mitochondrial translocation of Bax and Bak, depolarization of MMP, and subsequent release of cytochrome *c* and Smac (Figures 4 and 5). On the other hand, the downregulation of anti-apoptotic proteins, Bcl-xL and XIAP over time may contribute to JRS-15-induced stronger apoptosis (Figure 6). Furthermore, pre-treatment with caspase-9 or a pan-caspase inhibitor or Bcl-xL or XIAP overexpression all effectively prevented JRS-15-induced HeLa cell apoptosis (Figure 4C,D and Figure 6B–E). In addition, the proteolytic activity of initiator caspase-8, required for the extrinsic apoptotic pathway, was not detected under this apoptotic procession (Figure 4). Taken together, these results clearly demonstrate that JRS-15 induces HeLa cell apoptosis by triggering the intrinsic mitochondria-mediated caspase-9-dependent pathway. A proposed mechanism of JRS-15-induced apoptotic signaling has been illustrated in Figure 9.

Although both JRS-15 and xylocydine induce apoptosis via the intrinsic apoptosis pathway, there is still one question why JRS-15 is more potent than its parent xylocydine. Previous report has shown that xylocydine triggers apoptosis in HCC cell by inhibiting the activities of Cdk1, Cdk2, Cdk7 and Cdk9. However, JRS-15 losses the inhibitory effects on these Cdks (Figure S3), and the exact molecular target of JRS-15-induced apoptosis has not been identified yet in this study. Based on Lipinski’s “the rule of 5” and a knowledge-based approach in drug discovery and development, the AlogP value of drug-like between (−0.40) and 5.60 will be ideal [28]. According to this principle, JRS-15 with less polarity (AlogP = 2.85) may be more effective to across the cell membrane than xylocydine (AlogP = −1.53, polar) does, which partially explains the more potent anticancer activity of JRS-15.

Interestingly, the five cancer cell lines tested showed differential sensitivities to JRS-15 (Figure 2, Table 1). JRS-15 was more effective at inducing apoptosis in HeLa, HepG2, and A549 cells than in SK-HEP-1 and PC-3M cells (Figure 3E,F). Dysregulation of the apoptosis pathway is a common feature of cancer cells. Different cancer cells have specific expression patterns of apoptosis-regulating proteins [31,32]. In this study, JRS-15 showed quite different cell growth inhibition and pro-apoptotic effects in the two hepatoma cell lines, HepG2 and SK-HEP-1 (Figure 2, Table 1 and Figure 3E,F). We examined the expression patterns of apoptosis-regulating proteins in these two cell lines [33], and found that the expression level of caspase-9 was much lower in SK-HEP-1 than in HepG2 cells (Figure 8A). We then constructed a caspase-9 overexpressed SK-HEP-1 cell line (SK-Cas-9), and determined cell viability and caspase-3 activity upon treatment with JRS-15. SK-Cas-9 cells exhibited markedly increased sensitivity to JRS-15 in terms of cell viability and *in vitro* caspase-3 activity compared with SK-HEP-1 cells, and the viability trend was similar to that in HepG2 cells (Figure 8). These observations suggest that the reduced expression of caspase-9 in SK-HEP-1 cells may partially explain the mechanism of JRS-15 resistance, and restoration of caspase-9 expression is able to reestablish JRS-15-induced SK-HEP-1 cell apoptosis.

## 4. Experimental Section

### 4.1. Chemistry: General

The ^1^H and ^13^C NMR data were recorded with a Varian Mercury 300 NMR spectrometer, using TMS as an internal standard. Chemical shifts (δ) are given in parts per million and coupling constants are given as absolute values expressed in Hertz. Mass spectra were obtained using LC/MS 1100 of Agilent Technology Corporation and Alltech ELSD 2000 instrument. Melting points were determined by an X-4 digital microscope. Column chromatography was generally performed on silica gel (300–400 mesh) and TLC inspections on silica gel GF254 plates.

### 4.2. 4-Amino-6-(3-(3-Bromophenyl)Phenyl)-7-(β-l-Xylofuranosyl)Pyrolo[2,3-d]Pyrimidine-5-Cyano (JRS-15)

To a 25 mL round-bottom flask were charged **11** [30,34] (0.136 g, 0.20 mmol) and 3-bromophenyl boronic acid (0.060 g, 0.3 mmol) together with palladium tetrakis-triphenylphosphine (11.6 mg, 0.01 mmol) and K_2_CO_3_ (0.082 g, 0.6 mmol) in toluene (5.0 mL) under an atmosphere of N_2_. The reaction was heated at 100 °C for 22 h and cooled to room temperature. To this mixture was then added sequentially 3-bromophenyl boronic acid (0.048 g, 0.24 mmol) together with palladium tetrakis-triphenylphosphine (11.6 mg, 0.01 mmol) and K_2_CO_3_ (0.066 g, 0.48 mmol). The mixture was heated at 100 °C for another 22 h, then was diluted with water (10 mL) and extracted with ethyl acetate. The combined organic extracts were washed with brine, dried over MgSO_4_ and evaporated in vacuo. The crude product was purified by flash chromatography to give compound **12** as a yellow-white solid, using a solvent system of ethylacetate and chloroform (1:9, *v*/*v*).

The above obtained compound **12** was treated with NaOMe (16.00 mg, 0.30 mmol) in MeOH (5 mL) for 12 h at room temperature or until completion of the reaction as shown by TLC. The mixture was co-evaporated with silica and chromatographed using a solvent system of methanol and chloroform (1:9, *v*/*v*) on a column of silica to afford **JRS-15** (a white solid 0.044 g, 86% yield). mp 150–152 °C. IR (KBr): 3435(OH), 3331 (NH2) 2913, 2218 (CN), 1633, 1599. MS (ESI): 521.9 *m*/*z* [M + H^+^]. ^1^H NMR (300MHz, DMSO-d6): 8.31 (s, 1H), 7.93–7.99 (m, 3H), 7.76–7.81 (m, 2H), 7.62–7.70 (m, 2H), 7.48 (t, 1H, *J* = 7.4Hz), 7.19 (s, 2H, NH_2_), 6.69 (d, 1H, *J* = 6.0 Hz), 5.93 (d, 1H, *J* = 2.7 Hz), 5.51 (d, 1H, *J* = 2.1 Hz), 4.79–4.82 (m, 2H), 3.93–4.00 (m, 1H), 3.70–3.73 (m, 1H), 3.60–3.62 (m, 1H). Calcd for C_19_H_19_N_5_O_4_: C, 55.18; H, 3.86; N, 13.41. Found: C, 55.20; H, 3.84; N, 13.45.

### 4.3. Reagents and Antibodies

Xylocydine was supplied by College of Chemistry, Jilin University. Xylocydine and JRS-15 were dissolved in DMSO at a concentration of 50 mM as stock solution and stored at −80 °C. 3-(4,5-dimethylthiazol-2-yl)-2,5-diphenyltetrazoliumbromide (MTT), 4,6-diamidino-2-phenylindile (DAPI), propidium iodide (PI) were purchased from Sigma (St. Louis, MO, USA). The substrate Histone H1, caspase substrates Ac-DEVD-AFC, Ac-IETD-AFC, and Ac-LEHD-AFC, caspase inhibitors z-IETD-fmk, z-LEHD-fmk, and z-VAD-fmk, and the MitoCapture reagent were purchased from Calbiochem (La Jolla, CA, USA). The Mitochondria Isolation Kit was purchased from Pierce (Pierce, IL, USA). Antibodies against cytochrome *c*, PARP, Smac, c-IAP-1, c-IAP-2, XIAP, Bcl-xL, Bcl-2, Bak, Bax, c-Myc, Cdk1, Cdk2, β-actin, and α-tubulin were purchased from Santa Cruz Biotechnology (Santa Cruz, CA, USA). Antibodies against caspase-8, caspase-9, and Cox II were purchased from Cell Signaling Technology (Beverly, MA, USA). Antibodies against RNA polymerase II CTD (phospho S2) and RNA polymerase II CTD (phospho S5) were purchased from Abcam (Cambridge, UK).

### 4.4. Cell Culture and MTT Assay

HeLa, HepG2, SK-HEP-1, PC-3M, and A549 cells were maintained at 37 °C in 5% CO_2_ in Dulbecco’s Modified Eagle’s Medium (DMEM) (Invitrogen, Carlsbad, CA, USA) supplemented with 10% newborn calf serum (Invitrogen, Carlsbad, CA, USA) and 100 μg/mL penicillin, 100 μg/mL streptomycin (Invitrogen, Carlsbad, CA, USA). LO2 cells were maintained in Roswell Park Memorial Institute (RPMI) 1640 medium (Invitrogen, Carlsbad, CA, USA) supplemented with 10% fetal bovine serum (Invitrogen), the other cultured conditions were the same as described above. Cells were seeded in 96-well plates at 1 × 10^4^ cells/well in triplicate. After incubation for 24 h, cells were treated with indicated concentrations of JRS-15 or xylocydine. At 44 h post-treatment, 20 μL of MTT (5 mg/mL) was added to each well and incubated for 4 h. The formazan crystals formed by viable cells were solubilized with 200 μL of DMSO, and the color intensity was measured at 550 nm with an ELISA plate reader (BioTec Instruments, Winooski, VT, USA).

### 4.5. DAPI Staining Assay

HeLa cells were cultured for 24 h and treated or not with z-VAD-fmk for 2 h prior to treatment with 25 μM JRS-15 for 24 h. Cells were then fixed and stained with DAPI to visualize the nuclei and DNA. Images were captured under a fluorescence microscope (Olympus, Tokyo, Japan).

### 4.6. Cell Cycle Distribution Assay

HeLa cells were treated or not with the indicated concentrations of JRS-15 for 24 h or 48 h; LO2 cells were treated or not with the indicated concentrations of JRS-15 for 48 h, and fixed in ice-cold 75% ethanol at 4 °C overnight. Fixed cells were washed twice with PBS, stained with PI, and incubated on ice for 30 min in the dark. Cell cycle distribution was analyzed using a flow cytometry ((FACSCalibur, Becton-Dickinson, San Jose, CA, USA).

### 4.7. Annexin V-FITC/PI Double Staining Assay

HeLa cells were treated or not with 25 μM JRS-15 for 24 h and stained with Annexin V-FITC/PI. The percentage of Annexin V-FITC/PI-positive cells was determined by flow cytometry (FACSCalibur, Becton-Dickinson, San Jose, CA, USA).

### 4.8. Cell-Free Caspase Activity Assay

HeLa, HepG2, SK-HEP-1, PC-3M, A549, and LO2 cells were treated or not with 25 μM JRS-15 or 50 μM xylocydine for the indicated times. Following treatment, 50 μg cell lysates were incubated with 200 nM Ac-DEVD-AFC (for caspase-3), Ac-IETD-AFC (for caspase-8), and Ac-LEHD-AFC (for caspase-9) in a reaction buffer containing 20 mM HEPES pH 7.4, 100 mM NaCl, 10 mM DTT, 0.1% CHAPS, and 10% sucrose at 37 °C for 1 h. The reaction was monitored by fluorescence emission at 535 nm and excitation at 405 nm.

### 4.9. Immunoblot Analysis

Cells were washed with ice-cold PBS and solubilized in a RIPA lysis buffer containing a protease inhibitor cocktail (Roche, South San Francisco, CA, USA) and 1 mM PMSF. After incubation on ice for 1 h, the insoluble materials were removed by centrifugation at 12,000 rpm for 15 min. Equivalent amounts of total protein (50 μg or 100 μg) from each sample were analyzed by SDS-PAGE followed by electro-transfer to a PVDF membrane (Gelman, St. Louis, MO, USA). The membrane was blocked with 5% non-fat milk and probed with the indicated antibodies. The blots were washed and incubated with a horseradish peroxidase-coupled secondary antibody (Pierce), followed by detection by ECL assay (Amersham, Piscataway, NJ, USA).

### 4.10. Preparation of Mitochondrial and Cytosolic Protein Extracts

HeLa cells were treated or not with 25 μM JRS-15 for the indicated times. Mitochondrial and cytosolic protein extracts were prepared using a Mitochondria Isolation Kit (Pierce, Rockford, IL, USA) according to the manufacturer’s instructions.

### 4.11. Depolarization Assay of Mitochondrial Membrane Potential

HeLa cells were treated or not with 25 μM JRS-15 for the indicated times and incubated with 1.0 μg/mL of MitoCapture cation dye (Calbiochem, La Jolla, CA, USA) at 37 °C for 30 min. Images were captured by a fluorescence microscope (Olympus, Tokyo, Japan) with excitation wavelengths of 500 and 570 nm, respectively.

### 4.12. Transient Transfection Assay

HeLa cells were plated and then transfected using PolyFect Transfection Reagent (Qiagen, Valencia, CA, USA) according to the manufacturer’s instructions. Cells were co-transfected with 0.5 μg of pEGFP-N3 and 1.5 μg of pCS4 or pCS4-Bcl-xL or pCS4-XIAP for 24 h followed by an additional 24 h of treatment with JRS-15. Images were captured by a fluorescence microscope (Olympus, Tokyo, Japan).

### 4.13. Analysis the Value of AlogP

The AlogP values of JRS-15 and xylocydine were analysis by Accelrys Discovery Studio 2.5 (Accelrys, San Diego, CA, USA).

## 5. Conclusions

In conclusion, JRS-15 functions as a broad-spectrum anticancer agent in human cancer cells by blocking cell cycle and further triggering apoptosis via the intrinsic pathway. This leads to the translocation of Bax and Bak to the mitochondria, resulting in the depolarization of MMP and the release of cytochrome *c* and Smac, which is coincided with the downregulation of Bcl-xL and XIAP, thereby triggering the downstream caspase cascade. In addition, JRS-15 has less cytotoxic effect on normal cells, and is selective at inducing apoptosis between cancer and normal cells. Taken these together, JRS-15 is a potential candidate as a powerful chemotherapeutic agent. Furthermore, investigating the mechanisms of apoptosis resistance in certain cell lines may provide new solutions for cancer therapy.

## Supplementary Information



## Figures and Tables

**Scheme I:**
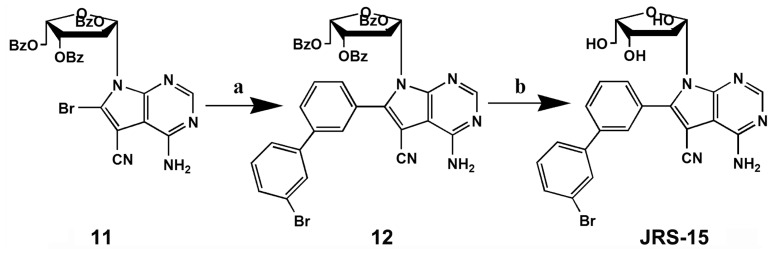
Reagent and condition: (**a**) Pd(PPh_3_)_4_, 3-bromophenyl boronic acid, K_2_CO_3_, toluene; (**b**) NaOCH_3_/CH_3_OH.

**Table 1:** The IC_50_ values were determined in various cell lines after 48 h of treatment with JRS-15.

Cell types	Cell line	JRS-15	Xylocydine

		IC_50_ (μM)	IC_50_ (μM)
Cervical carcinoma	HeLa	12.50	Exceeding 50
Hepatic carcinoma	HepG2	12.42	Exceeding 50
	SK-HEP-1	28.25	Exceeding 50
Prostate carcinoma	PC-3M	27.20	Exceeding 50
Lung adenocarcinoma	A549	14.25	Exceeding 50
Normal liver cell line	LO2	Exceeding 50	Exceeding 50
