# Supplementary material for: Apoptosis is Induced in Cancer Cells via the Mitochondrial Pathway by the Novel Xylocydine-Derived Compound JRS-15
Source: Int J Mol Sci. 2013 Jan 4;14(1):850–70. doi: 10.3390/ijms14010850 (PMC3565295; doi:10.3390/ijms14010850)
Supplement: Supplementary file 1 [file ijms-14-00850-s001.pdf]

Supplementary Information

Figure S1. Xylocydine does not activate the caspases even at a concentration of 75 μM of treatment. HeLa cells were treated with 0.15% DMSO (*v*/*v*) (Control) or with 75 μM xylocydine for the indicated times. (**A**–**C**) Cell-free caspase-3, -8 and -9 activities were measured. (**D**) Whole-cell lysates were analyzed by immunoblotting for caspase-8, caspase-9, PARP and β-actin.

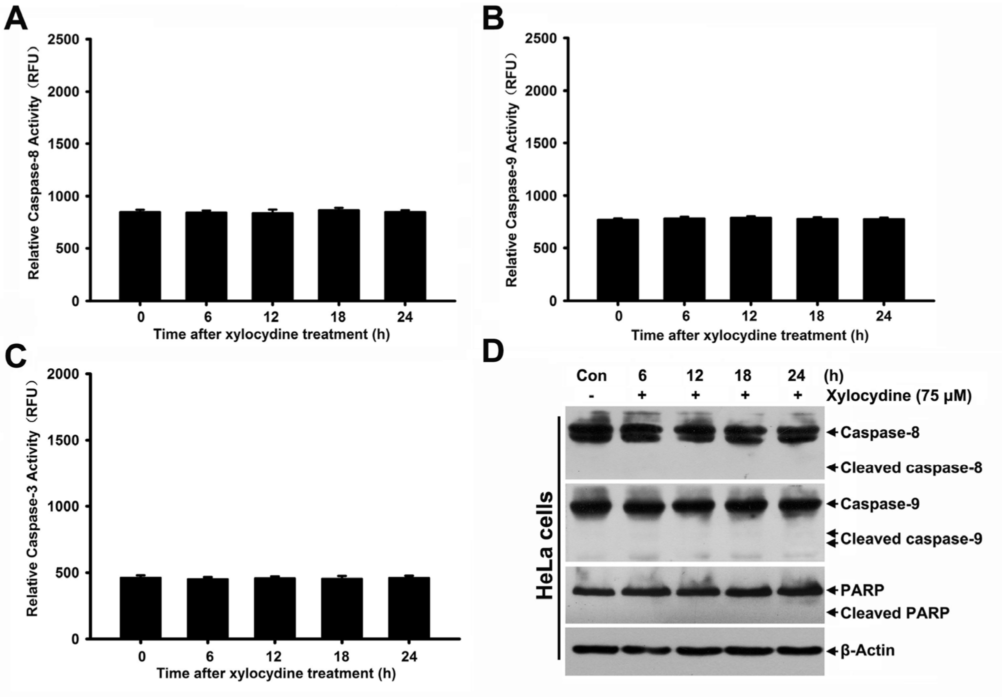

Figure S2. JRS-15 does not induce the cleavages of caspases and PARP in LO2 cells at a concentration of 30 μM of treatment. LO2 cells were treated with 0.1% DMSO (*v*/*v*) (Control) or with 30 μM JRS-15 for the indicated times. Whole-cell lysates were analyzed by immunoblotting for caspase-8, caspase-9, PARP and β-actin.

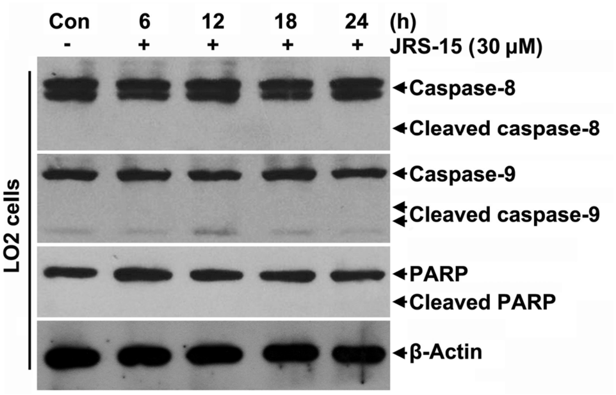

Figure S3. JRS-15 cannot inhibit Cdk1 and Cdk2 activity *in vitro*, as well as Cdk7 and Cdk9 activity in cultured HeLa cells. (**A**) The kinase assays were performed at 30 °C for 15 min in the reaction buffer (50 mM Tris-HCl (pH 7.4), 10 mM $MgCl_2$, 1 mM EGTA, 50 mM β-glycerophosphate, 0.1 mM Na_3VO_4, 25 mM NaF, 2 mM DTT, protease inhibitors and 0.1 mM phenylmethylsulfonyl fluoride) with a final volume of 50 µl that containing 5 µg Histone H1 protein as a specific substrate, Cdk1/cyclin B or Cdk2/cyclin A immunoprecipitated from protein extract of HeLa cells as an enzyme source, 10 µCi of [γ-^{32}P]ATP, 15 µM ATP and 10 µM compound. Samples were analyzed by 12% SDS-PAGE followed by autoradiography. The compound of number 15 represents JRS-15, and xylocydine (xyc.) as a positive control. (**B**) HeLa cells were treated with the indicated concentrations of JRS-15 for 24 h. Whole-cell lysates were analyzed for Cdk7/9-specific phosphorylation of RNA polymerase II-CTD by immunoblotting, β-actin as a loading control.

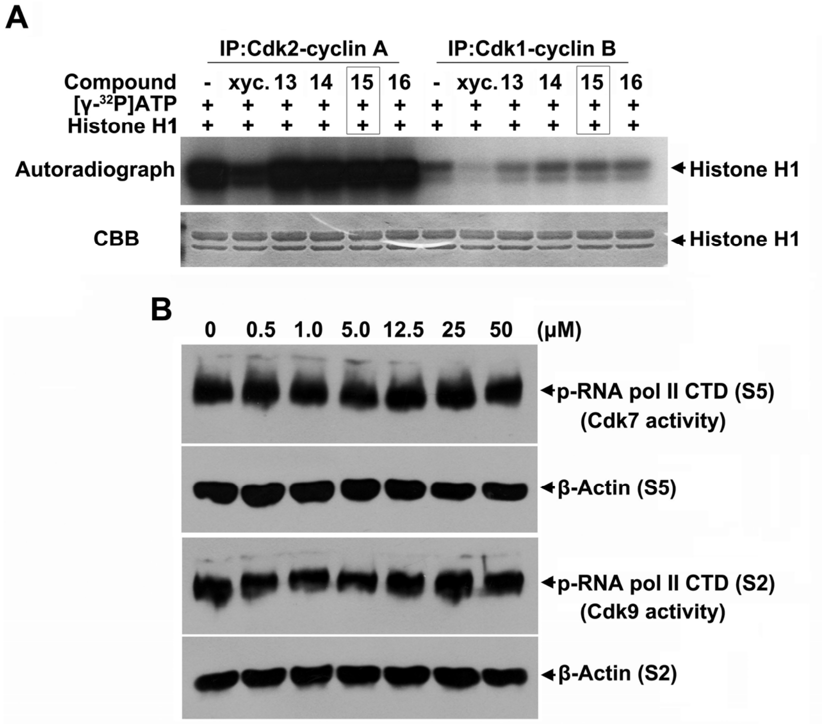

© 2013 by the authors; licensee MDPI, Basel, Switzerland. This article is an open access article distributed under the terms and conditions of the Creative Commons Attribution license (http://creativecommons.org/licenses/by/3.0/).
